# Supplementary material for: Dynalign II: common secondary structure prediction for RNA homologs with domain insertions
Source: Nucleic Acids Res. 2014 Nov 21;42(22):13939–48. doi: 10.1093/nar/gku1172 (PMC4267632; doi:10.1093/nar/gku1172)
Supplement: SUPPLEMENTARY DATA [file supp_gku1172_nar-02021-z-2014-File012.zip › manual/Overview/Installation_Instructions_Mac.html]

RNAstructure Installation and Overview: Installation Instructions for Macintosh Systems


|  |  |  |
| --- | --- | --- |
|  | RNAstructure Installation and Overview Installation Instructions for Macintosh Systems | - Contents - Index |
| This page details how to install the pre-built package. For information on how to build the package, please refer to the Building Instructions and Building Requirements pages.  To install the JAVA graphical user interface (GUI), follow these steps:   1. Make sure JAVA 1.6 or higher is installed on your system. Apple now has its own version of JAVA, use the "Software Update" function of your Mac to make sure JAVA is up to date. "Software update" is found as an item in the "System preferences." 2. Download the JAVA GUI from the Mathews Lab RNAstructure page. 3. The GUI is supplied as a GZipped tarball, so the files must be extracted. Move the tarball to the desired directory and double click. 4. A directory called RNAstructure will now be in the same directory as the tarball. The tarball can be deleted if desired. 5. Double-click the RNAstructure directory. Then double-click the RNAstructure icon. You will be asked if you want to run a program downloaded from the internet; click "open." The examples directory within RNAstructure provides sample input files. 6. A note on **Mac OS X Mountain Lion**: When attempting to run the Java GUI on Mountain Lion, an error message may occur that says the executable is damaged. This error message is misleading, and actually means that security settings must be changed to run the GUI. If this message appears, follow these steps: Click on "System Properties." Go into "Security & Privacy." Click on the lock to allow changes to be made. Select "Anywhere" under "Allow applications downloaded from:." 7. Note that the RNAstructure app can be dragged to the application folder, if desired, or kept where the package was extracted. 8. The online help for the GUI is available here. It can also be found under the help menu item from within the program.   To install the text interfaces, follow these steps:   1. Download the text interfaces for Mac from the Mathews Lab RNAstructure page. 2. The text interfaces are supplied as a GZipped tarball, so the files must be extracted. Move the tarball to the desired directory and execute the command *tar -xzvf RNAstructure\_tar\_file\_name*. 3. A folder called RNAstructure will now be in the same directory as the tarball. The tarball can be deleted if desired. 4. The text interface executables are in the RNAstructure/exe directory, and can be run directly from there. 5. Before running the text interfaces, please refer to command line help page for specifying the location of the thermodynamic parameters. 6. The online help for the command line programs is available here. | | |
| Visit The Mathews Lab RNAstructure Page for updates and latest information. | | |
